# Oral therapies for treatment of relapsing–remitting multiple sclerosis in Austria: a 2-year comparison using an inverse probability weighting method

**DOI:** 10.1007/s00415-020-09811-6

**Published:** 2020-04-03

**Authors:** Michael Guger, Christian Enzinger, Fritz Leutmezer, Jörg Kraus, Stefan Kalcher, Erich Kvas, Thomas Berger, the Austrian MS Treatment Registry (AMSTR)

**Affiliations:** 1 Clinic for Neurology 2, Kepler University Hospital GmbH, Med Campus III, Krankenhausstr. 9, 4021 Linz, Austria; 2 Medical Faculty, Johannes Kepler University Linz, Linz, Austria; 3 Department of Neurology, Medical University of Graz, Graz, Austria; 4 Department of Neurology, Medical University of Vienna, Vienna, Austria; 5 Department of Laboratory Medicine, Paracelsus Medical University and Salzburger Landeskliniken, Salzburg, Austria; 6 Department of Neurology, Medical Faculty, Heinrich-Heine-University, Düsseldorf, Germany; 7 Hermesoft, Data management, Graz, Austria; 8 Hermesoft, Statistics, Graz, Austria

**Keywords:** Comparison, Dimethyl fumarate, Fingolimod, Inverse probability weighting, Multiple sclerosis, Teriflunomide

## Abstract

**Objectives:**

To compare the efficacies, frequencies and reasons for treatment interruption of fingolimod (FTY), dimethyl fumarate (DMF) or teriflunomide (TERI) in a nationwide observational cohort.

**Materials and methods:**

Two cohorts of patients with relapsing–remitting multiple sclerosis (RRMS) having started treatment with FTY, DMF or TERI documented in the Austrian MS Treatment Registry (AMSTR) since 2014 and either staying on therapy for at least 24 months (24 m cohort) or with at least one follow-up visit after start of treatment (total cohort). The 24 m cohort included 629 RRMS patients: 295 in the FTY, 227 in the DMF and 107 in the TERI group. We used multinomial propensity scores for inverse probability weighting in generalized linear and Cox proportional hazards models to correct for the bias of this non-randomised registry study.

**Results:**

Estimated mean annualized relapse rates (ARR) over 24 months were 0.13 for FTY, 0.09 for DMF and 0.11 for TERI treatment. For TERI in comparison with DMF, we observed higher probability for treatment interruption (*p* = 0.023) and reduced sustained EDSS regression for 12 (*p* = 0.016) and 24 weeks (*p* = 0.031) and, for the comparison of DMF versus FTY, a reduced sustained EDSS progression for 12 weeks (*p* = 0.02).

**Conclusions:**

Relapse rates with treatment with FTY, DMF and TERI were similar. Patients treated with DMF showed less sustained disability progression for 12 weeks than FTY-treated patients. However, FTY and DMF treatment was associated with more likely EDSS regression for 12 and 24 weeks and a lower probability for treatment interruption as compared to TERI-treated patients.

**Electronic supplementary material:**

The online version of this article (10.1007/s00415-020-09811-6) contains supplementary material, which is available to authorized users.

## Introduction

Treatment efficacy of fingolimod (FTY), dimethyl fumarate (DMF) and teriflunomide (TERI) for relapsing remitting multiple sclerosis (RRMS) has been proven in randomised trials [1–6]. In comparison to placebo groups, FTY reduced the annualized relapse rate (ARR) by 48–54% [1, 2], DMF by 44–53% [3, 4] and TERI by 32–36% [5, 6]. In addition, FTY showed a reduction of the ARR by 52% versus interferon beta-1a [7]. In post hoc comparisons of DMF versus glatiramer acetate, differences were not significant except for new and/or enlarging T2-weighted hyperintense lesions [4]. No difference in ARR between TERI and IFNβ-1a was seen in the TENERE study [8].

Studies matching the clinical efficacy provided conflicting results [9–20]. These discrepancies ask for further investigations to confirm or rebut the published findings, especially by real-life experiences.

The objective of our study was, first, to compare the efficacy of FTY, DMF or TERI and, second, to analyse the probability for stopping, pausing or switching (treatment interruption) of these therapies in a nationwide observational cohort using prospectively collected data from a real-life setting.

## Materials and methods

### Data collection

The Austrian MS Treatment Registry (AMSTR) [20, 21], established in 2006 to maintain quality control and comply with reimbursement regulations of the Austrian sick funds, allows to obtain clinical data, to assess indications, the clinical profiles of the treated patients and to monitor safety in real life. The AMSTR is part of the dense MS network in Austria, which is constituted by all MS clinics from neurological departments and some dedicated neurological doctoral offices. In addition, prescriptions of DMTs for MS are exclusively restricted to MS centers. Thus, prescriptions and treatment documentations are evenly distributed across Austria. The AMSTR is compliant with Austrian laws on bioethics and was approved by the ethical committee of the Medical University of Vienna (EC number 2096/2013).

AMSTR documents anonymous baseline data, including MS onset and duration, relapses in the prior 12 months, EDSS, gross MRI activity and previous disease-modifying therapies (DMT). Follow-up data (relapses, EDSS, adverse events [AE’s], change or discontinuation of treatment) are required to be documented every 3–6 months, median visit interval 3.8 months for fingolimod, 4 months for DMF and 3.8 months for teriflunomide. Each relapse had to be confirmed by a neurologist at the MS center and documented in the AMSTR. Documentation required relapse onset, EDSS and use/dosage of i.v. methylprednisolone treatment. Besides the fact that applying the AMSTR is mandatory for reimbursement, a special quality-related feature of the AMSTR is an external and independent data monitoring to improve data management in terms of completeness and plausibility of documented data.

In 2011, the European Medicines Agency (EMA) approved FTY along the same indication criteria as natalizumab. Reimbursement for FTY in Austria adheres to this approval. Thus, FTY-treated patients in Austria had to have either at least one relapse in the prior 12 months despite treatment with interferon beta or glatiramer acetate and at least 9 T2 lesions or at least one Gadolinium enhancing lesion on recent brain MRI (“indication A”), or two or more severe relapses in the preceding treatment-naïve 12 months and one or more Gadolinium enhancing lesions on brain MRI or a significant increase in T2 lesion load as compared to a previous recent MRI (“indication B”).

In 2013, TERI and in 2014, DMF were approved by the EMA with the indication for the treatment of adult patients with RRMS.

We investigated a total cohort of 1530 patients, who started treatment with FTY, DMF or TERI in the AMSTR at any time since 2014. The coverage of the AMSTR for the three oral agents is approximately 70% of total prescription in Austria. For the purpose of this study, we analysed the data of these patients in two separate cohorts. The first cohort stayed on therapy for at least 24 months (24 m cohort), and this group was analysed for comparing the efficacies of the different oral drugs. The second cohort was the total cohort, defined by availability of at least one follow-up visit, also including the 24 m cohort. This group was analysed for the frequency, cause and risk of interruption (total cohort).

The primary outcome measure was the ARR during treatment with FTY, DMF or TERI over 2 years after initiation of therapy. Relapses were defined as new or worsening neurological symptoms lasting for at least 24 h in the absence of fever.

Further outcome measures were the total number of relapses, EDSS progression or regression confirmed after 12 and 24 weeks, and EDSS changes during the 2-year period (difference between EDSS at the last visit and at baseline). Sustained disability progression or regression was defined as an increase or decrease from baseline of at least 1.0 point in the EDSS score (or at least 0.5 points for patients with a baseline EDSS score greater than 5.5) that persisted for at least 12 or 24 weeks.

For analyses of the treatment interruption, we defined three causes, namely (a) stopping treatment as permanent treatment interruption in the AMSTR; (b) pausing treatment as treatment interruption and restarting with the same treatment; and (c) switching treatment as treatment interruption and starting with a new medication in the AMSTR.

### Statistical methods

All effects estimated in comparing treatment groups were average treatment effects (ATE). To control the bias for non-randomised assignment to the treatment groups, we used inverse probability weighting (IPW) and propensity score (PS) matching as a comparison method. When comparing three groups, we used the estimation of multinomial propensity scores as described by McCaffrey [22]. Propensity scores for treatment with FTY, DMF and TERI were estimated for all patients with the baseline parameters age, duration of disease, number of relapses 12 months prior to baseline, EDSS, presence of at least 9 MRI T2 lesions and at least one contrast-enhancing MRI lesion, and previous therapy as independent variables. These variables were included in the model because of their clinical meaning, independent from their significance as a predictor in the model. Therefore, we tried to overcome the problems of being misled by false positive predictors in a multiple testing situation as well as missing relevant variables by abandoning them in a beta failure decision. Treatment groups were balanced for all variables after scoring (Table S1). Our PS estimations for IPW were optimized for the Kolmogorov–Smirnov (KS) statistic, because this method compares the entire distribution rather than just the mean.

A generalized linear model (GLM) with relapse count as Poisson-distributed dependent variable and log transformed observation time in years as offset variable was used to estimate the treatment effect on the ARR in the 24 months observation period. To overcome a potential immortal time bias, we secondary analysed ARR in an observation period without a time limit.

Augmented inverse probability weighting was used to analyse the change of EDSS from baseline to the last visit in the 24 months observation period, so the mean differences between last visit and baseline (negative as improvement, positive as worsening) could be estimated for each treatment from the potential means generated by the model.

We used Cox proportional hazards models for analysing EDSS progression and regression confirmed after 12 and 24 weeks, and the relapse hazard in the 24 months observation period.

Cox proportional hazards models were also used analysing treatment interruptions in the patient cohort with at least one follow-up visit.

All models included treatment as categorical factor and inverse multinomial propensity scores as weights regarding the survey character of the study. All variables used for propensity scoring were also used in the outcome models as independent variables to obtain adjusted treatment effects. We applied this double robust approach, because the ATE estimator remains consistent if at least one of the two, the propensity score model or the outcome regression, is specified properly. Thus, the misspecification of only one of the two models would not cause any harm to the ATE estimator [23].

For all Cox models, the proportional hazards assumption had been verified by non-significant deviations from the proportional hazards assumption using Schoenfeld residuals.

As statistical programmes, we used IBM SPSS Statistics for Windows, Version 24.0 (Armonk, NY: IBM Corp.), Stata Statistical Software, Release 15 (College Station, TX: StataCorp LP.), R package twang version 1.5.

## Results

The 24-month continuous treatment cohort included 629 RRMS patients: 295 in the FTY, 227 in the DMF, and 107 in the TERI group. The baseline data of the 629 patients are summarized in Table 1 and show certain imbalances for some baseline variables. IPW resulted in a weighted sample size of 1508 patients: 551 in the FTY, 545 in the DMF, and 412 in the TERI group (Tables S2 and S3). The number of patients interrupting treatment within 24 months was 99 for FTY treatment (48 stopped, 12 paused and 39 switched), 93 for DMF (45 stopped, 8 paused and 40 switched) and 54 for TERI (18 stopped, 3 paused and 33 switched).

**Table 1:** Baseline patient characteristics of the 24 months continuous treatment cohort

	FTY *N* = 295 46.9%	DMF *N* = 227 36.1%	TERI *N* = 107 17.0%	Total *N* = 629 100%
Female				
* N*	191	155	64	410
%	64.7%	68.3%	59.8%	65.2%
Age*				
Mean	39.5	38.1	42.8	39.6
SD	10.6	10.6	9.9	10.6
Duration of MS at baseline (years)*				
Mean	9.5	8.0	8.8	8.9
SD	7.4	8.7	8.1	8.0
EDSS at baseline*				
Mean	2.5	1.7	2.1	2.1
SD	1.6	1.2	1.4	1.5
Relapse rate within 12 months prior treatment start*				
Mean	1.3	1.0	0.7	1.1
SD	0.8	0.8	0.7	0.8
Prior treatment**				
Yes				
* N*	271	127	67	465
%	91.9%	55.9%	62.6%	73.9%
No				
*N*	24	100	40	164
%	8.1%	44.1%	37.4%	26.1%
≥ 9 T2 lesions				
Yes				
* N*	268	196	89	553
%	90.8%	86.3%	83.2%	87.9%
No				
* N*	27	31	18	76
%	9.2%	13.7%	16.8%	12.%
≥ 1 Gd-enhancing T1 lesion**				
Yes				
* N*	153	94	38	285
%	51.9%	41.4%	35.5%	45.3%
No				
* N*	142	133	69	344
%	48.1%	58.6%	64.5%	54.7%
Indication***				
A				
* N*	185	0	0	185
%	62.7%	0.0%	0.0%	62.7%
B				
* N*	110	0	0	110
%	37.3%	0.0%	0.0%	37.3%
Follow-up in months				
Mean	24.3	24.2	24.4	24.3
SD	1.0	1.1	1.1	1.1

*DMF* dimethylfumarate, *EDSS*, expanded disability status scale, *FTY* fingolimod, *Gd* gadolinium, *MS* multiple sclerosis, *SD* standard deviation, *TERI* teriflunomide

*Comparison using Kruskal–Wallis test revealed *p *value < 0.05

**Comparison using Chi quadrat test revealed *p *value < 0.05

***Indication A, at least one relapse in the prior 12 months despite treatment with either interferon beta or glatiramer acetate; indication B, at least two severe relapses in the prior 12 months in treatment-naive patients

Estimated mean annualized relapse rates (ARR) from the GLM were 0.13 for FTY (95% CI 0.04–0.43) over 24.2 months (95% CI 24.1–24.4), 0.09 for DMF (95% CI 0.03–0.26) over 24.3 months (95% CI 24.1–24.4) and 0.11 for TERI treatment (95% CI 0.04–0.35) over 24.6 months (CI 24.3–24.9), leading to incidence rate ratios (IRR) of 1.43 for FTY versus DMF (95% CI 0.92–2.22, *p* = 0.110) and 1.21 for TERI versus DMF (95% CI 0.69–2.12, *p* = 0.512). Analysing ARR from the GLM in an observation period without a time limit results were similar, no significant differences were observed between treatments. Finally, PS matching produced differences between treatments similar in comparison with IPW, but also without statistical significance.

Estimated mean relapse counts from the GLM within the first 3 months were 0.11 for FTY (95% CI 0.01–0.99), 0.09 for DMF (95% CI 0.01–0.8) and 0.03 for TERI (95% CI 0.002–0.63), leading to IRR of 1.18 for FTY versus DMF (95% CI 0.50–2.80, *p* = 0.708) and 0.37 for TERI versus DMF (95% CI 0.08–1.81, *p* = 0.221).

**Fig. 1:**
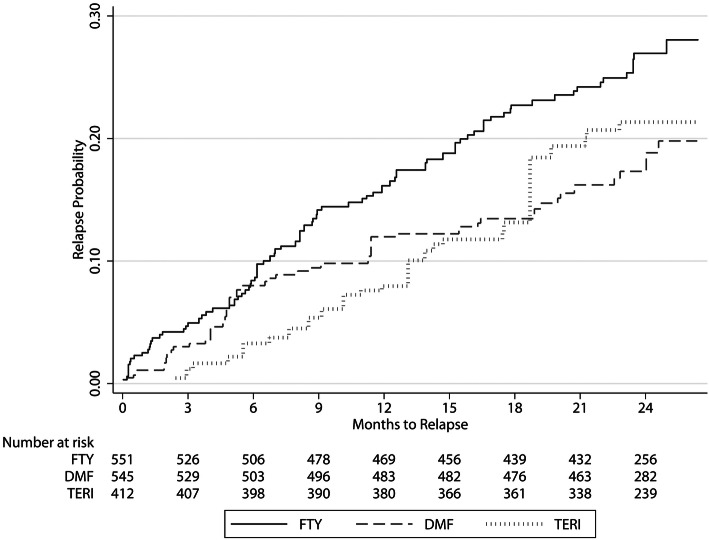
Cumulative probability for experiencing a relapse within the first 24 months of RRMS treatment with fingolimod, dimethyl fumarate or teriflunomide. *DM*F dimethyl fumarate, *FTY* fingolimod, *TERI* teriflunomide

80 patients treated with FTY (27.1%) experienced a relapse in the 24 months period, and respective frequencies were 40 (17.6%) for those treated with DMF and 23 (21.5%) for TERI, with an estimated HR of 1.34 for FTY versus DMF (95% CI 0.85–2.10, *p* = 0.202) and 1.27 for TERI versus DMF (95% CI 0.66–2.43, *p* = 0.478) (Fig. 1).

Mean EDSS change in the FTY group was − 0.002 (95% CI − 0.13 to 0.13) versus − 0.127 for DMF (95% CI − 0.23 to − 0.03) with a difference between treatments (FTY vs DMF) of 0.126 (95% CI − 0.04 to 0.29, *p* = 0.136), 0.123 for TERI (95% CI − 0.10 to 0.35) versus − 0.127 for DMF (95% CI − 0.23 to − 0.03) with a difference between treatments (TERI vs DMF) of 0.251 (95% CI 0.004–0.5, *p* = 0.047) and -0.002 for FTY (95% CI − 0.13 to 0.13) versus 0.123 for TERI (95% CI − 0.10 to 0.35) with a difference between treatments (FTY vs TERI) of 0.125 (95% CI − 0.387 to 0.137, *p* = 0.351).

**Fig. 2:**
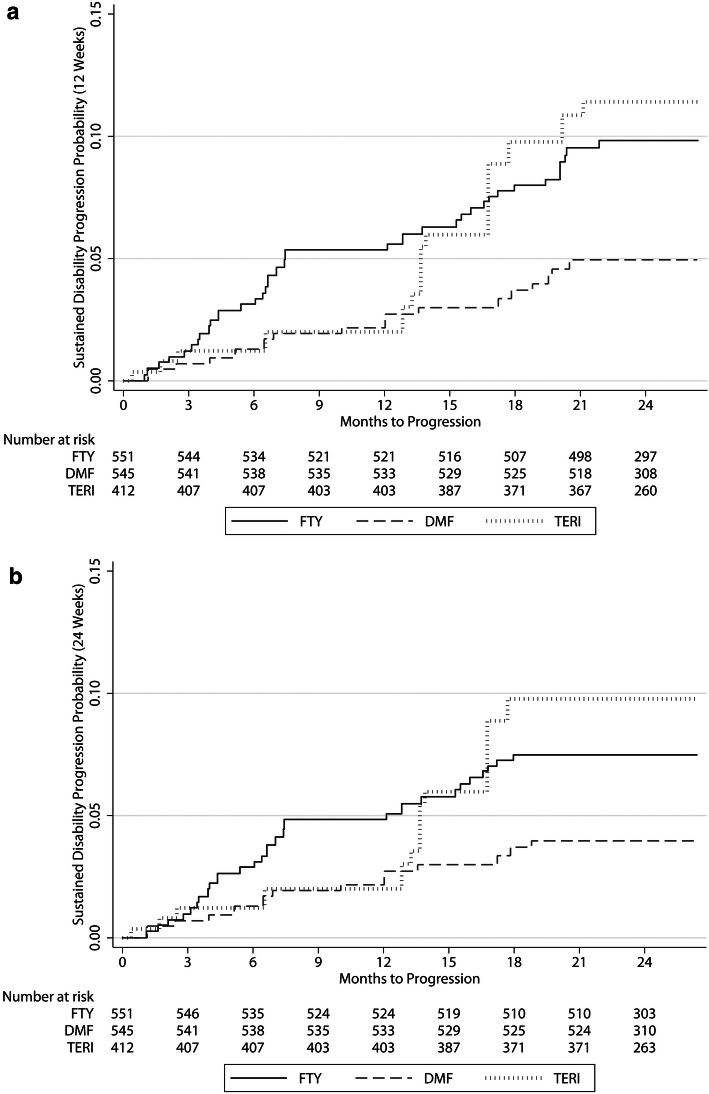

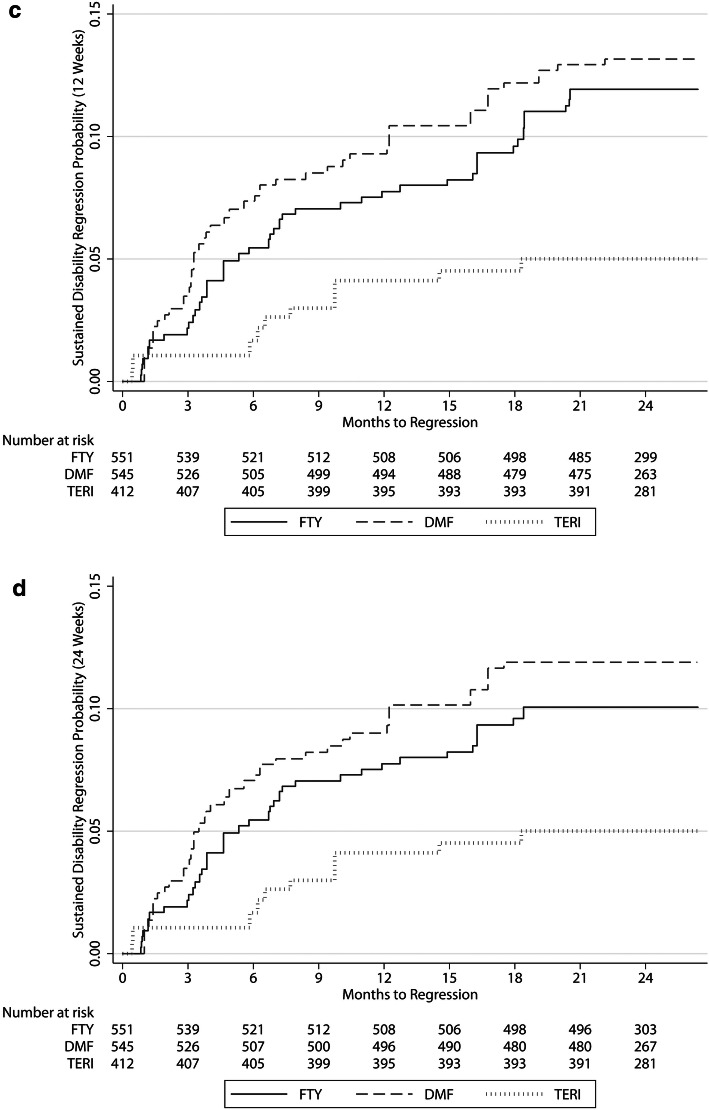
**a**, **b** Cumulative probability for disability progression sustained for 12 (**a**) and 24 weeks (**b**) within the first 24 months of RRMS treatment with fingolimod, dimethyl fumarate or teriflunomide. **c**, **d** Cumulative probability for disability regression sustained for 12 (**c**) and 24 weeks (**d**) within the first 24 months RRMS treatment with fingolimod, dimethyl fumarate or teriflunomide. *DMF* dimethyl fumarate, *FTY* fingolimod, *TERI* teriflunomide

Sustained EDSS progression for 12 weeks was significantly different between FTY and DMF (HR 2.23, 95% CI 1.14–4.38; *p* = 0.020), and a trend in the same direction was observed concerning sustained EDSS progression for 24 weeks for FTY versus DMF (HR 1.99, 95% CI 0.94–4.2; *p* = 0.071). There were no significant differences regarding sustained EDSS progression for 12 weeks and 24 weeks between TERI and DMF (HR 2.26, 95% CI 0.93–5.47; *p* = 0.071 and HR 2.47, 95% CI 0.92–6.64; *p* = 0.074) (Fig. 2a, b), with a trend towards reduced EDSS progression with DMF. During the first year of follow-up, 12 DMF and 9 TERI patients showed EDSS progression for 12 and 24 weeks. From month 12 to 18, EDSS progression was more pronounced for TERI, resulting in 32 TERI and 8 DMF patients with EDSS progression for 12 and 24 weeks.

Sustained EDSS regression for 12 and 24 weeks comparing FTY versus DMF was not different (HR 0.79, 95% CI 0.44–1.41; *p* = 0.417 and HR 0.72, 95% CI 0.39–1.34; *p* = 0.298), comparing TERI versus DMF the differences were significant (HR 0.34, 95% CI 0.14–0.82; *p* = 0.016 and HR 0.38, 95% CI: 0.16–0.92; *p* = 0.031) (Fig. 2c, d).

The total cohort comprised 1530 RRMS patients (585 with FTY, 651 with DMF and 294 with TERI). Baseline data are summarized in Table 2 and show a certain imbalance for some baseline variables. For analysing hazard ratios for treatment interruption, again inverse probability weighting was used, resulting in a weighted sample size of 3998 patients (1327 in the FTY, 1423 in the DMF, and 1248 in the TERI group) (Table S4).

**Table 2:** Baseline patient characteristics of the total cohort

	FTY *N* = 585 38.2%	DMF *N* = 651 42.5%	TERI *N* = 294 19.2%	Total *N* = 1530 100%
Female				
* N*	395	443	189	1027
%	67.5%	68.0%	64.3%	67.1%
Age*				
Mean	39	38	43	39
SD	11	11	10	11
Duration of MS at baseline (years)*				
Mean	9.3	6.8	8.4	8.0
SD	7.6	8.1	7.7	7.9
EDSS at baseline*		.		
Mean	2.4	1.6	2.0	2.0
SD	1.6	1.3	1.4	1.5
Relapse rate within 12 months prior treatment start*				
Mean	1.4	1.0	0.7	1.1
SD	0.9	0.8	0.7	0.8
Prior treatment**				
Yes				
*N*	510	336	189	1035
%	87.2%	51.6%	64.3%	67.6%
No				
* N*	75	315	105	495
%	12.8%	48.4%	35.7%	32.4%
≥ 9 T2 lesions**				
Yes				
* N*	527	544	248	1319
%	90.1%	83.6%	84.4%	86.2%
No				
* N*	58	107	46	211
%	9.9%	16.4%	15.6%	13.8%
≥ 1 Gd-enhancing T1 lesion**				
Yes				
* N*	302	294	100	696
%	51.6%	45.2%	34.0%	45.5%
No				
* N*	283	357	194	834
%	48.4%	54.8%	66.0%	54.5%
Indication***				
A				
* N*	369	0	0	369
%	63.1%	0.0%	0.0%	63.1%
B				
* N*	216	0	0	216
%	36.9%	0.0%	0.0%	36.9%
Follow-up in months*				
Mean	31.8	21.6	28.4	26.8
SD	17.5	14.2	17.1	16.7

*DMF* dimethyl fumarate, *EDSS* expanded disability status scale, *FTY* fingolimod, *Gd* gadolinium, *MS* multiple sclerosis, *SD* standard deviation, *TERI* teriflunomide

*Comparison using Kruskal–Wallis test revealed *p *value < 0.05

**Comparison using Chi quadrat test revealed *p *value < 0.05

***Indication A, at least one relapse in the prior 12 months despite treatment with either interferon beta or glatiramer acetate; indication B, at least two severe relapses in the prior 12 months in treatment-naive patients

**Fig. 3:**
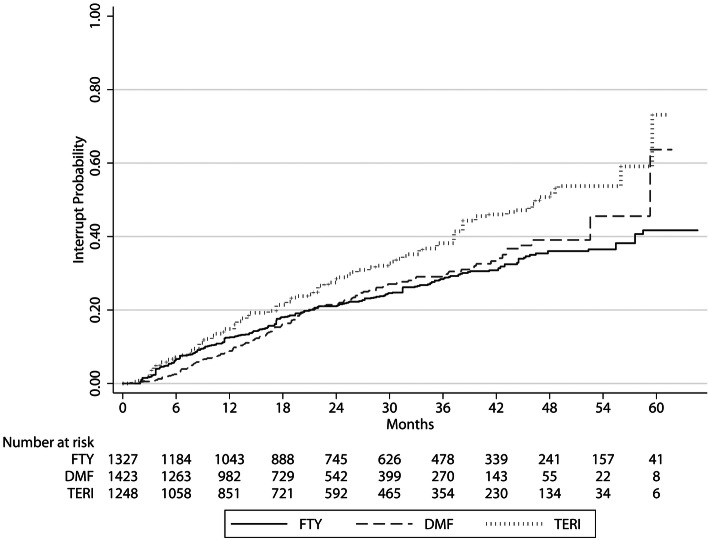
Cumulative probability for treatment interruption. *DMF* dimethyl fumarate, *FTY* fingolimod, *TERI* teriflunomide

The hazard ratios for treatment interruption comparing FTY versus DMF were 0.96 (95% CI 0.72–1.27; *p* = 0.764) and 1.42 comparing TERI versus DMF (95% CI 1.05–1.91), *p* = 0.023 (Fig. 3).

The number of patients interrupting treatment was 158 (27%) for FTY treatment (83 stopped, 13 paused and 62 switched), 135 (20.7%) for DMF (71 stopped, 11 paused and 53 switched) and 95 (32.3%) for TERI (39 stopped, 4 paused and 52 switched).

62 patients switched from FTY (10.2%), namely 25 to natalizumab, 17 to DMF, 8 to alemtuzumab, 6 to TERI, 3 to cladribine, 2 to ocrelizumab or 1 to daclizumab. 53 patients switched from DMF (8.1%), namely 27 to FTY, 13 to natalizumab, 5 to TERI, 4 to cladribine, 3 to alemtuzumab or 1 to ocrelizumab and finally 52 patients from TERI (17.7%), 27 to FTY, 14 to DMF, 7 to natalizumab, 3 to ocrelizumab or 1 to cladribine.

The mean time period until treatment interruption was 19.7 months (SD 13.8) for FTY, 18 months (SD 11.8) for DMF and 20.6 months (SD 14.8) for TERI.

The reasons for interrupting FTY were mainly patients’ wishes (patient’s decision) (*n* = 92), disease progression (clinical and/or radiological activity; *n* = 68) and adverse events (AEs) (*n* = 64), for DMF patients’ wishes (*n* = 91), AEs (*n* = 51) and disease progression (*n* = 43). The main reasons for interrupting TERI were disease progression (*n* = 54) followed by patients’ wishes (*n* = 50) and AEs (*n* = 34). Pregnancy or the wishes to conceive were documented in 12 patients in the FTY, 20 patients in the DMF and 3 patients in the TERI cohort. Treating neurologists were allowed to name several reasons per patient.

The ARR for patients staying on treatment over the whole observation period (26.8 months, SD 16.7) was 0.18 (SD 0.49) for FTY, 0.18 (SD 0.58) for DMF and 0.10 (SD 0.34) for TERI and for patients with treatment interruption 0.39 (SD 0.71) under FTY, 0.54 (SD 0.99) under DMF and 0.75 (SD 1.78) under TERI until interruption.

The ARR after switching to another treatment or restarting after a treatment interruption stayed low in all treatment groups (FTY 0.13 (SD 0.34), DMF 0.42 (SD 0.96) and TERI 0.41 (SD 1.31). Mean wash-out period or treatment pause were 3.5 (SD 4.8) months for FTY, 3.5 (SD 4.8) months for DMF and 4 (SD 6.9) months for TERI. Mean observation period after treatment switch or restart was 17.6 (SD 14.3) months for FTY, 12.6 (SD 10.2) months for DMF and 17.8 (SD 14.4) months for TERI.

## Discussion

In this observational study, we prospectively collected data to compare the efficacy of FTY, DMF and TERI in 629 patients who continuously received treatment for at least 24 months, and in a wider population of 1530 patients who had at least one follow-up visit subsequent to starting therapy.

The different approved indications caused differences in the cohorts at baseline (Table 1). In particular, the TERI group was older and less likely to have had a relapse in the prior 12 months. Over 90% of the FTY patients had received prior treatment as compared to only 56% of the DMF and 63% of the TERI cohort. In contrast, DMF patients were younger and less disabled with shorter disease duration.

To account and control for these documented differences, we used inverse probability weighting (IPW) and as a comparison method propensity score matching (PS). To demonstrate balance or imbalance after matching we optimized our PS estimations for IPW for the Kolmogorov–Smirnov (KS) statistic (Tables S2 and S3). In comparison of both, IPW and PS, the differences regarding ARR were not significant.

On the basis of these results and as three treatments needed to be compared, we used the method of IPW instead of propensity score (PS) matching. One reason for that lies within the fact that PS matching would have generated three different two-group comparisons (FTY-DMF; TERI-DMF; FTY-TERI). This would have produced different subpopulations for each treatment group in its particular comparison to the other two treatment groups, depending on the PS overlap and the following matching result. Here we saw the risk of comparing patients with the lowest scores in the treatment group with patients showing the highest scores in the control group. Besides losing information of unmatched patients, we thus would have run risk to compare patients atypical for respective treatments with patients who might be considered atypical for the control treatment. Furthermore, IPW offers opportunity to use all patients of our populations avoiding the problem of missing data, also allowing for considering all three treatments at once with the chosen models.

As a further measure to reduce bias, we decided to use all variables of the PS model also in the outcome models, leading to further adjustment for the treatment effects.

Comparing our present results with prior published 12 months’ data [20], we found a significantly higher EDSS impairment, lower EDSS regression and a higher interruption rate in the TERI group. The longer observation period on treatment (at least 24 months) produced more robust data especially in regards to disease progression and regression.

Two previous studies also compared between these oral MS drugs [16, 17]. Ontaneda et al., analysed patients from a commercial claims database, switching from platform disease-modifying therapies (DMTs) to DMF, FTY and TERI and staying on treatment for at least 3 months. Comparable post-index ARR were observed between DMF and FTY, but were significantly lower with DMF versus TERI [16]. In contrast, Kalincik et al. [17] showed a lower ARR on FTY compared with DMF and TERI analysing 614 (TERI), 782 (DMF) or 2332 (FTY) patients from the global MSBase cohort, staying at least 3 months on treatment. No differences in disability accumulation or improvement were found between these therapies.

In contrast to the aforementioned study, our whole study population had to be on treatment for at least 24 months, leading to an overall lower ARR rate and possibly resulting in more robust and comparable data. In addition, we used the method of IPW instead of propensity score matching.

The hazard ratio for treatment interruption comparing TERI versus DMF and FTY was significantly higher.

The main reason for interrupting FTY and DMF were adverse events and patients’ wishes, but for TERI, clearly disease progression, resulted in a higher switching rate in the TERI cohort as compared to FTY- and DMF-treated patients.

These results are in contrast to Vollmer et al. [10], who found a lower discontinuation rate for FTY (34.3%) versus DMF (47.1%), driven by adverse events. Hersh et al. [12] also reported a higher likelihood of early discontinuation of DMF (41.3% versus 35.6%), mostly again due to adverse events.

Kalincik et al. [17] observed lower discontinuation rates (24% with DMF and TERI and 10% with FTY), and lack of efficacy was relatively more commonly reported in TERI and DMF patients in comparison with FTY.

Immortal time bias is a problem in studies comparing a treatment group with a minimum survival time as qualification condition to a control group without this limit. In our study, this qualification condition was given for all groups. In advance, we compared the interrupt frequency between the treatment groups and observed comparable interrupt rates in the first 24 months for the observed reasons switch, pause and stop. Also the time until these events were comparable. Differences in ARR were only observed in single highly active patients in the early phase of the disease. Analyzing ARR in an observation period without a time limit results were similar, no significant differences were observed. In this evaluation, FTY showed the lowest ARR, followed by DMF and TERI. The reason for the lower ARR in the FTY cohort was based on the fact that FTY patients had longer observation periods than the DMF and TERI groups resulting in fewer relapses in the later phase of the disease. Finally we tried to avoid immortal time bias analyzing EDSS progression/regression confirmed at 3 and 6 months, which would be induced allowing short observation periods.

In summary, we believe the minimum qualification time should not produce a relevant bias for the comparison between the three treatment groups.

The strengths of our study are that this work represents data from a nationwide observational study, comprising patients in Austria who have been treated with FTY, DMF and TERI since 2014. The AMSTR is a secure web-based platform, which enables treating neurologists in all Austrian MS centres to immediately perform online documentation during patient visits. To ensure high documentation and data quality in terms of completeness and plausibility, the AMSTR is monitored by an external and independent clinical research organization. This real world data shows a low ARR, progression rate and discontinuation rate for all three oral drugs reflecting high quality maintenance of MS patients in Austria.

As an important limitation of our study, MRI data were only available at baseline before starting treatment with FTY, DMF and TERI and were included as an independent variable for propensity scoring and in the respective outcome models.

In conclusion, we found no difference analysing ARR and probability for experiencing a relapse between the three oral treatment regimen, but there were significant differences regarding (1) EDSS impairment, higher rates of treatment interruption and reduced sustained EDSS regression for 12 and 24 weeks comparing TERI with DMF and (2) reduced sustained EDSS progression for 12 weeks concerning DMF versus FTY.

## Electronic supplementary material

Below is the link to the electronic supplementary material.

Supplementary file1 (DOCX 20 kb)

Supplementary file2 (DOCX 14 kb)

Supplementary file3 (DOCX 84 kb)

Supplementary file4 (DOCX 81 kb)
