# Supplementary material for: Oral therapies for treatment of relapsing–remitting multiple sclerosis in Austria: a 2-year comparison using an inverse probability weighting method
Source: J Neurol. 2020 Apr 3;267(7):2090–100. doi: 10.1007/s00415-020-09811-6 (PMC7320928; doi:10.1007/s00415-020-09811-6)
Supplement: Supplementary file 1 — Supplementary file1 (DOCX 20 kb) [file 415_2020_9811_MOESM1_ESM.docx]

Table S1. Minimum p-values for all pairwise group comparisons between FTY, DMF, TERI per variable before and after weighting with inverse propensity scores.

| Variable | minimum p-value  unweighted | minimum p-value  weighted |
| --- | --- | --- |
| 24 months continuous treatment cohort |  |  |
| Age | 0.0000 | 0.9732 |
| Duration of MS at baseline | 0.0003 | 0.5648 |
| Relapse rate within 12 months prior treatment start | 0.0000 | 0.5005 |
| EDSS at baseline | 0.0001 | 0.4877 |
| ≥ 9 T2 lesions | 0.0319 | 0.5172 |
| ≥ 1 Gd-enhancing T1 lesion | 0.0040 | 0.1586 |
| Prior treatment | 0.0000 | 0.0668 |
| total cohort |  |  |
| Age | 0.0000 | 0.9188 |
| Duration of MS at baseline | 0.0000 | 0.3250 |
| Relapse rate within 12 months prior treatment start | 0.0000 | 0.2330 |
| EDSS at baseline | 0.0000 | 0.4512 |
| ≥ 9 T2 lesions | 0.0008 | 0.3278 |
| ≥ 1 Gd-enhancing T1 lesion | 0.0000 | 0.1544 |
| Prior treatment | 0.0000 | 0.0677 |

DMF = dimethylfumarate; EDSS = Expanded Disability Status Scale; FTY = fingolimod; Gd = gadolinium; MS = multiple sclerosis; TERI = teriflunomide
