# Supplementary material for: Oral therapies for treatment of relapsing–remitting multiple sclerosis in Austria: a 2-year comparison using an inverse probability weighting method
Source: J Neurol. 2020 Apr 3;267(7):2090–100. doi: 10.1007/s00415-020-09811-6 (PMC7320928; doi:10.1007/s00415-020-09811-6)
Supplement: Supplementary file 2 — Supplementary file2 (DOCX 14 kb) [file 415_2020_9811_MOESM2_ESM.docx]

Table S2. Balance of our covariates of the 24 months continuous treatment cohort using Kolmogorov Smirnov statistic

| FTY vs. DMF | KS | p-value |
| --- | --- | --- |
| Age | 0.0485 | 0.9699 |
| Duration of MS at baseline | 0.0692 | 0.7272 |
| Relapse rate within 12 months prior treatment start | 0.0690 | 0.7302 |
| EDSS at baseline | 0.0838 | 0.4935 |
| ≥ 9 T2 lesions | 0.0207 | 1.0000 |
| ≥ 1 Gd-enhancing T1 lesion | 0.0726 | 0.6725 |
| Prior treatment | 0.0827 | 0.5101 |
| FTY vs. TERI | KS | p-value |
| Age | 0.0442 | 1.0000 |
| Duration of MS at baseline | 0.1161 | 0.5611 |
| Relapse rate within 12 months prior treatment start | 0.1214 | 0.5048 |
| EDSS at baseline | 0.0865 | 0.8705 |
| ≥ 9 T2 lesions | 0.0009 | 1.0000 |
| ≥ 1 Gd-enhancing T1 lesion | 0.0147 | 1.0000 |
| Prior treatment | 0.1334 | 0.3889 |
| DMF vs. TERI | KS | p-value |
| Age | 0.0530 | 0.9991 |
| Duration of MS at baseline | 0.0565 | 0.9977 |
| Relapse rate within 12 months prior treatment start | 0.0731 | 0.9629 |
| EDSS at baseline | 0.0826 | 0.9088 |
| ≥ 9 T2 lesions | 0.0216 | 1.0000 |
| ≥ 1 Gd-enhancing T1 lesion | 0.0579 | 0.9969 |
| Prior treatment | 0.0506 | 0.9996 |

DMF = dimethylfumarate; EDSS = Expanded Disability Status Scale; FTY = fingolimod; Gd = gadolinium; KS = Kolmogorov Smirnov; MS = multiple sclerosis; TERI = teriflunomide
