# Supplementary material for: Oral therapies for treatment of relapsing–remitting multiple sclerosis in Austria: a 2-year comparison using an inverse probability weighting method
Source: J Neurol. 2020 Apr 3;267(7):2090–100. doi: 10.1007/s00415-020-09811-6 (PMC7320928; doi:10.1007/s00415-020-09811-6)
Supplement: Supplementary file 3 — Supplementary file3 (DOCX 84 kb) [file 415_2020_9811_MOESM3_ESM.docx]

Table S3. Baseline patient characteristics of the 24 months continuous treatment cohort after inverse probability weighting

|  |  | |  |  |  | SMD |  | Test |
| --- | --- | --- | --- | --- | --- | --- | --- | --- |
|  | FTY  (N=551) | | DMF  (N=545) | TERI  (N=412) | FTY-DMF | DMF-TERI | FTY-TERI | FTY-DMF-TERI |
| Age | |  |  |  |  |  |  |  |
| Mean | | 39,5 | 39,4 | 39,5 | 0,0089 | -0,0095 | 0,0010 | GLM p=0.976 |
| SD | | 12 | 10,7 | 7,8 |  |  |  |  |
|  | |  |  |  |  |  |  |  |
| Duration of MS at baseline (years) | |  |  |  |  |  |  |  |
| Mean | | 9,4 | 8,8 | 7,7 | 0,0656 | 0,1431 | 0,2187 | GLM p=0.109 |
| SD | | 8,8 | 8,8 | 5,6 |  |  |  |  |
|  | |  |  |  |  |  |  |  |
| Relapse rate within 12 months prior treatment start | |  |  |  |  |  |  |  |
| Mean | | 1,2 | 1,1 | 0,9 | 0,1460 | 0,2197 | 0,3633 | GLM p=0.007 |
| SD | | 0,9 | 0,8 | 0,5 |  |  |  |  |
|  | |  |  |  |  |  |  |  |
| EDSS at baseline | |  |  |  |  |  |  |  |
| Mean | | 2,3 | 2 | 2 | 0,1722 | 0,0060 | 0,1906 | GLM p=0.051 |
| SD | | 1,7 | 1,3 | 0,9 |  |  |  |  |
|  | |  |  |  |  |  |  |  |
| ≥ 9 T2 lesions | |  |  |  |  |  |  |  |
| N | | 495 | 478 | 370 | 0,1168 | -0,1218 | -0,0051 | chi² p=0.756 |
| % | | 89,8% | 87,7% | 89,9% |  |  |  |  |
|  | |  |  |  |  |  |  |  |
| ≥ 1 Gd-enhancing T1 lesion | |  |  |  |  |  |  |  |
| N | | 272 | 229 | 197 | 0,1624 | -0,1301 | 0,0324 | chi² p=0.484 |
| % | | 49,3% | 42,0% | 47,8% |  |  |  |  |
|  | |  |  |  |  |  |  |  |
| Prior treatment | |  |  |  |  |  |  |  |
| N | | 437 | 387 | 272 | 0,2453 | 0,1285 | 0,3737 | chi² p=0.117 |
| % | | 79,3% | 71,0% | 66,0% |  |  |  |  |

chi² = Chi Quadrat test; DMF = dimethylfumarate; EDSS = Expanded Disability Status Scale; FTY = fingolimod; Gd = gadolinium; GLM = generalized linear model; MS = multiple sclerosis; SMD = Standardized mean differences; SD = standard deviation; TERI = teriflunomide
