# Supplementary material for: Oral therapies for treatment of relapsing–remitting multiple sclerosis in Austria: a 2-year comparison using an inverse probability weighting method
Source: J Neurol. 2020 Apr 3;267(7):2090–100. doi: 10.1007/s00415-020-09811-6 (PMC7320928; doi:10.1007/s00415-020-09811-6)
Supplement: Supplementary file 4 — Supplementary file4 (DOCX 81 kb) [file 415_2020_9811_MOESM4_ESM.docx]

Table S4. Baseline patient characteristics of the total cohort after inverse probability weighting

|  |  |  |  |  | SMD |  | Test |
| --- | --- | --- | --- | --- | --- | --- | --- |
|  | FTY  (N=1327) | DMF  (N=1423) | TERI  (N=1248) | FTY-DMF | DMF-TERI | FTY-TERI | FTY-DMF-TERI |
| Age |  |  |  |  |  |  |  |
| Mean | 39,5 | 39,2 | 39,4 | 0,0209 | -0,0171 | 0,0039 | GLM p=0.916 |
| SD | 10,5 | 10,9 | 10,6 |  |  |  |  |
|  |  |  |  |  |  |  |  |
| Duration of MS at baseline (years) |  |  |  |  |  |  |  |
| Mean | 8,4 | 7,8 | 7,6 | 0,0794 | 0,0272 | 0,1109 | GLM p=0.151 |
| SD | 7,6 | 8 | 7,3 |  |  |  |  |
|  |  |  |  |  |  |  |  |
| Relapse rate within 12 months prior treatment start |  |  |  |  |  |  |  |
| Mean | 1,2 | 1,1 | 1 | 0,1114 | 0,1076 | 0,2155 | GLM p=0.017 |
| SD | 0,8 | 0,8 | 0,8 |  |  |  |  |
|  |  |  |  |  |  |  |  |
| EDSS at baseline |  |  |  |  |  |  |  |
| Mean | 2,1 | 1,9 | 1,9 | 0,1112 | 0,0137 | 0,1280 | GLM p=0.074 |
| SD | 1,5 | 1,4 | 1,3 |  |  |  |  |
|  |  |  |  |  |  |  |  |
| ≥ 9 T2 lesions |  |  |  |  |  |  |  |
| N | 1158 | 1210 | 1050 | 0,1034 | 0,0379 | 0,1413 | chi² p=0.536 |
| % | 87,2% | 85,1% | 84,1% |  |  |  |  |
|  |  |  |  |  |  |  |  |
| ≥ 1 Gd-enhancing T1 lesion |  |  |  |  |  |  |  |
| N | 655 | 633 | 549 | 0,1083 | 0,0106 | 0,1189 | chi² p=0.337 |
| % | 49,3% | 44,5% | 44,0% |  |  |  |  |
|  |  |  |  |  |  |  |  |
| Prior treatment |  |  |  |  |  |  |  |
| N | 965 | 945 | 807 | 0,1644 | 0,0432 | 0,2076 | chi² p=0.102 |
| % | 72,7% | 66,4% | 64,6% |  |  |  |  |

chi² = Chi Quadrat test; DMF = dimethylfumarate; EDSS = Expanded Disability Status Scale; FTY = fingolimod; Gd = gadolinium; GLM = generalized linear model; MS = multiple sclerosis; SMD = Standardized mean differences; SD = standard deviation; TERI = teriflunomide
